# Does psychological distress predict risk of orthopaedic surgery and postoperative opioid prescribing in patients with hip pain? A retrospective study

**DOI:** 10.1186/s12891-024-07418-w

**Published:** 2024-04-20

**Authors:** Kent F. Sutton, Grant H. Cabell, Lucas W. Ashley, Trevor A. Lentz, Brian D. Lewis, Steven A. Olson, Richard C. Mather

**Affiliations:** 1 Duke University School of Medicine, Duke University Medical Center, Durham, NC USA; 2 Department of Orthopaedic Surgery, Duke University Medical Center, Durham, NC USA; 3 Brody School of Medicine, East Carolina University, Greenville, NC USA

**Keywords:** Psychological distress, Hip pain, Orthopaedics, Opioids, Function, Quality of life, Public health

## Abstract

**Background:**

Clinicians and public health professionals have allocated resources to curb opioid over-prescription and address psychological needs among patients with musculoskeletal pain. However, associations between psychological distress, risk of surgery, and opioid prescribing among those with hip pathologies remain unclear.

**Methods:**

Using a retrospective cohort study design, we identified patients that were evaluated for hip pain from January 13, 2020 to October 27, 2021. Patients’ surgical histories and postoperative opioid prescriptions were extracted via chart review. Risk of hip surgery within one year of evaluation was analyzed using multivariable logistic regression. Multivariable linear regression was employed to predict average morphine milligram equivalents (MME) per day of opioid prescriptions within the first 30 days after surgery. Candidate predictors included age, gender, race, ethnicity, employment, insurance type, hip function and quality of life on the International Hip Outcome Tool (iHOT-12), and psychological distress phenotype using the OSPRO Yellow Flag (OSPRO-YF) Assessment Tool.

**Results:**

Of the 672 patients, *n* = 350 (52.1%) underwent orthopaedic surgery for hip pain. In multivariable analysis, younger patients, those with TRICARE/other government insurance, and those with a high psychological distress phenotype had higher odds of surgery. After adding iHOT-12 scores, younger patients and lower iHOT-12 scores were associated with higher odds of surgery, while Black/African American patients had lower odds of surgery. In multivariable analysis of average MME, patients with periacetabular osteotomy (PAO) received opioid prescriptions with significantly higher average MME than those with other procedures, and surgery type was the only significant predictor. Post-hoc analysis excluding PAO found higher average MME for patients undergoing hip arthroscopy (compared to arthroplasty or other non-PAO procedures) and significantly lower average MME for patients with public insurance (Medicare/Medicaid) compared to those with private insurance. Among those only undergoing arthroscopy, older age and having public insurance were associated with opioid prescriptions with lower average MME. Neither iHOT-12 scores nor OSPRO-YF phenotype assignment were significant predictors of postoperative mean MME.

**Conclusions:**

Psychological distress characteristics are modifiable targets for rehabilitation programs, but their use as prognostic factors for risk of orthopaedic surgery and opioid prescribing in patients with hip pain appears limited when considered alongside other commonly collected clinical information such as age, insurance, type of surgery pursued, and iHOT-12 scores.

## Background

The opioid crisis remains a global public health emergency, especially in the United States, where 80,816 people died from opioid overdoses in 2021 alone [1]. Clinicians and public health professionals have prioritized research efforts to better understand the driving forces of opioid over-prescription, reduce consumption, and mitigate opioid-related risks [2–4]. Musculoskeletal conditions are a leading cause of disability, affecting over 1.71 billion people worldwide [5]. With increasing demand for orthopaedic evaluation, intervention, and pain management, it is not surprising that orthopaedic surgeons are the third-highest opioid prescribers (6–7). Opioids remain a fundamental component of postoperative pain management for orthopaedic surgery, creating an opportunity for diversion and abuse [8–10]. Opioid over-prescription and overuse among orthopaedic patients in the postoperative setting is well-documented [11–14]. Dose and duration of opioid prescriptions in the first postoperative month are critical determinants of chronic opioid use [15–17].

For the orthopaedic surgeon, patient-reported pain is a focal variable when considering the potential harms and benefits of surgery, as patients with higher-rated pain are more likely to be high priority candidates for surgery [18]. Patients exhibiting severe expressions of pain-associated psychological distress, such as pain catastrophizing, have underscored the need to develop treatment pathways that better address pain-related psychological factors [19]. Psychological distress and psychiatric comorbidities have been associated with higher rates of healthcare utilization, making patients susceptible to invasive procedures and extended hospitalizations [20–24]. Equally important are the implications of pain-associated psychological distress for postoperative opioid prescribing, as persistent opioid therapy is most common among patients with chronic musculoskeletal non-cancer pain [25].

There is a dearth of research investigating the relationship between multidimensional psychological distress, risk of surgery, and postoperative opioid prescribing among orthopaedic patients, especially those undergoing hip surgery. Evaluation and management of psychological distress and quality of life is becoming more common in orthopaedics with the development of tools such as the Optimal Screening for Prediction of Referral and Outcome Yellow Flag Assessment Tool (OSPRO-YF) and International Hip Outcome Tool (iHOT-12) [26–30]. However, these tools remain underutilized, and their potential applications are not fully understood. Classifying patients into clinically-relevant groups based on psychological distress constructs (i.e., “psychological phenotyping”) has been implemented in several orthopaedic studies, most recently in patients with hip pathologies [31–34]. A comprehensive understanding of multidimensional psychological distress in patients with hip pain who are at-risk of invasive procedures and opioid exposure may better inform treatment [2, 4, 32, 35–36].

The first aim of this study tested the following question: (1) Does patients’ level of psychological distress predict risk of undergoing hip surgery within one year of evaluation after controlling for hip-specific quality of life scores and demographic factors? This study’s second aim investigated the following question: (2) Does psychological distress predict opioid prescribing patterns in the first 30 days after surgery after controlling for hip-specific quality of life scores and demographic factors? We hypothesized that patients with more severe pain-associated psychological distress would have greater odds of undergoing hip surgery and receiving opioid prescriptions with higher dosages in the acute postoperative period.

## Methods

### Study design, setting, and participants

A retrospective cohort study design was used to identify patients evaluated in clinic by three hip orthopaedic surgeons (BDL, SAO, RCM) at a quaternary academic medical center. The study population included patients referred to hip preservation surgeons for hip pain by providers in addition to self-referred patients. Eligibility criteria included individuals aged 18–65 evaluated in clinic between January 13, 2020 and October 27, 2021. Participants without complete demographic and questionnaire data were excluded. Patients’ surgical encounters within one year of evaluation (aim one) and postoperative opioid prescriptions within 30 days of surgery (aim two) were ascertained via chart review.

### Description of experiment, variables, data sources, bias, & statistical analysis

Demographics including age, gender, race, ethnicity, employment, and health insurance were collected at patients’ initial orthopaedic visits. Participants completed the OSPRO-YF and iHOT-12 questionnaires at the beginning of each new patient visit. The OSPRO-YF evaluates general and pain-associated psychological distress among people with musculoskeletal conditions. This 10-item tool measures 11 unique psychological constructs, categorized into three domains (corresponding constructs in parentheses): negative mood (depression, trait anxiety, and trait anger); negative coping (fear-avoidance for work, fear-avoidance for physical activity, pain catastrophizing, kinesophobia, and pain anxiety); and positive affect/coping (pain self-efficacy, self-efficacy for rehabilitation, and pain acceptance) [26, 37]. The OSPRO-YF has good reliability and predictive validity for a variety of patient-reported outcomes among people with musculoskeletal pain [26, 38–40]. The OSPRO-YF has been used to identify common psychological phenotypes among people with hip pain [34]. Phenotypes include high distress, negative pain coping, low distress, and low self-efficacy and acceptance. For the purposes of this analysis, we assigned patients to their most likely psychological phenotype using previously described latent class analysis methods [34]. The iHOT-12 is a validated instrument that evaluates patient function and hip-related quality of life in four domains: symptoms and functional limitations; job-related concerns; sports and recreational activities; and social, emotional, and lifestyle concerns [27]. The iHOT-12 scores range from 0 to 100, with a score of 100 indicating superior quality of life and function [27].

### Aim one: predictive model for risk of surgery in one year

Participants’ surgical encounters were obtained via retrospective chart review. Hip surgeries were classified as hip arthroscopy, hip arthroplasty, combined periacetabular osteotomy (PAO) and hip arthroscopy, or other hip joint surgery. A predictive model was carried out with multivariable logistic regression to determine significant predictors of having surgery within one year of evaluation. We took a two-step approach to model development to better understand the unique contributions of psychological distress and function/quality of life for predicting these outcomes. We were particularly interested in whether the consideration of phenotypes, rather than raw OSPRO-YF scores, would be helpful in predicting outcomes. Assignment of phenotypes may be easier to interpret clinically than raw OSPRO-YF scores and more ecologically valid than the use of arbitrary cut-off scores. In the first step, candidate predictors in the model included age, gender, race, ethnicity, employment, insurance type, and OSPRO-YF phenotype assignment. In the second step, we added iHOT-12 scores to learn whether pain-associated psychological distress remained a significant predictor after accounting for hip-related function and quality of life information. Age and iHOT-12 scores were continuous variables. Race, ethnicity, employment, insurance type, and OSPRO-YF phenotype were categorical variables.

### Aim two: predictive model for mean MME of opioid prescriptions

For this aim, patients who underwent hip surgery within one year of evaluation with complete prescription data within 30 days of surgery were eligible for inclusion. Opioids prescribed in the first 30 postoperative days were included in our analyses. We developed a two-step multivariable regression model for average morphine milligram equivalents (MME) per day of opioid prescriptions, inclusive of refills. Average MME per day was a continuous variable calculated by computing a total MME per day using all opioid prescriptions for each patient and dividing by the patient’s total number of prescriptions. Standardized beta coefficients and p-values are reported for each predictive model. Tests of model effect for average MME of opioid prescriptions prescribed for patients were conducted with the Wald Chi-Square test and ANOVA.

Statistical significance was determined with a two-sided p-value < 0.05. IBM SPSS Statistics for Windows, Version 27.0 (IBM Corp., Armonk, NY, USA) and Latent GOLD software version 6.0 (Statistical Innovations, Belmont, MA, USA) were used to perform all statistical analyses. The study protocol was approved by the Duke University Health System Institutional Review Board (Durham, NC, USA).

## Results

### Aim one: predictive model for risk of surgery in one year

A total of 672 patients with complete questionnaire and follow-up data were included in the analytic dataset. Of the 672, *n* = 350 (52.1%) underwent hip surgery within one year of their baseline visit (Table 1). Patients most commonly pursued hip arthroscopy (*n* = 220), followed by hip arthroplasty (*n* = 85), PAO (*n* = 38), and other hip joint surgery (*n* = 7). Examples of other hip joint surgery included hip core decompression and girdlestone procedure. In the first model excluding iHOT-12 scores, logistic regression yielded three significant predictors of having surgery: age, insurance type, and OSPRO-YF phenotype assignment (Table 2). Younger patients (beta=-0.032, *p* < 0.001) and those with TRICARE/other government insurance (compared to private insurance, beta = 0.661, *p* = 0.005) had higher odds of undergoing hip surgery (Table 2). Patients assigned to the low distress (beta=-0.876, *p* < 0.001) and low self-efficacy and acceptance (beta=-0.592, *p* = 0.037) phenotypes had lower odds of undergoing surgery than patients assigned to the high distress phenotype (Table 2). In the second model that added iHOT-12 scores as a covariate, logistic regression yielded three significant predictors of surgery: age, race, and iHOT-12 score (Table 3). Older patients (beta=-0.035, *p* < 0.001) and Black/African American patients (beta=-0.659, *p* = 0.011) had lower odds of undergoing hip surgery compared to younger and White patients, respectively (Table 3). Furthermore, lower mean iHOT-12 scores (worse function) were associated with higher odds of undergoing surgery (beta=-0.041, *p* < 0.001) (Table 3). OSPRO-YF phenotype was not significant in this model.

**Table 1:** Participant demographics, surgical histories, and OSPRO-YF phenotype clusters (*n* = 721)

Age	
Mean (SD)	39.2 (13.1) years
**Gender**	
Male	272 (37.7%)
Female	449 (62.3%)
**Race**	
White	560 (77.7%)
Black or African American	95 (13.2%)
Asian	19 (2.6%)
American Indian or Alaskan Native	3 (0.4%)
Native Hawaiian or Other Pacific Islander	1 (0.1%)
Other	7 (1.0%)
Not reported/Declined	36 (5.0%)
**Ethnicity**	
Hispanic/Latino	36 (4.4%)
Not hispanic/Latino	653 (90.6%)
**Employment**	
Full time	332 (46.0%)
Part time	22 (3.1%)
Student–full time	52 (7.2%)
Student–Part Time	2 (0.3%)
Self employed	35 (4.9%)
Disabled	41 (5.7%)
Not employed	82 (11.4%)
Retired	34 (4.7%)
Unknown	44 (6.1%)
On active military duty	76 (10.5%)
**Insurance**	
Private	490 (68.0%)
Public	88 (12.2%)
TRICARE/other government	130 (18.0%)
Other	13 (1.8%)
**Surgery within one year**	
Yes	350 (48.5%)
No	367 (50.9%)
Unknown	4 (0.6%)
**OSPRO-YF phenotype cluster**	
High distress	299 (41.5%)
Negative pain coping	176 (24.4%)
Low distress	172 (23.9%)
Low self-efficacy & acceptance	74 (10.2%)

**Table 2:** Logistic regression analysis of predictors of surgery within one year excluding iHOT-12 scores (*n* = 672)

Predictor	Beta coefficient	P-value
Age	-0.032	< 0.001
Female gender (Ref: Male)	-0.041	0.819
Race (Ref: White)		
- Black/African American	-0.474	0.054
- Asian	-0.307	0.537
- Not reported	0.642	0.406
- Other	-0.131	0.803
Ethnicity (Ref: Not Hispanic/Latino)		
- Hispanic/Latino	-0.152	0.729
- Not reported	-0.479	0.328
Employment (Ref: Employed)		
- Not employed	-0.213	0.394
- Student	-0.074	0.821
- Other/Disabled	0.584	0.159
Insurance (Ref: Private)		
- Public	-0.008	0.981
- TRICARE/other government	0.661	0.005
- Other/Worker’s Compensation/Self pay	0.695	0.275
OSPRO-YF phenotype cluster (Ref: high distress)		
- Negative pain coping	-0.237	0.261
- Low distress	-0.876	< 0.001
- Low self-efficacy and acceptance	-0.592	0.037

**Table 3:** Logistic regression analysis of predictors of surgery within one year including iHOT-12 scores (*n* = 672)

Predictor	Beta coefficient	P-value
Age	-0.035	< 0.001
Female gender (Ref: Male)	0.250	0.187
Race (Ref: White)		
- Black/African American	-0.659	0.011
- Asian	-0.507	0.336
- Not reported	0.919	0.267
- Other	0.003	0.995
Ethnicity (Ref: not hispanic/Latino)		
- Hispanic/Latino	-0.282	0.539
- Not reported	-0.354	0.512
Employment (Ref: Employed)		
- Not employed	-0.169	0.520
- Student	0.205	0.559
- Other/Disabled	0.162	0.708
Insurance (Ref: Private)		
- Public	-0.380	0.272
- TRICARE/other government	0.351	0.159
- Other/Worker’s Compensation/Self pay	0.174	0.791
OSPRO-YF phenotype cluster (Ref: High Distress)		
- Negative pain coping	0.070	0.755
- Low distress	-0.036	0.886
- Low self-efficacy and acceptance	0.111	0.725
iHOT-12 Scores	-0.041	< 0.001

#### Aim two: predictive model for mean MME of opioid prescriptions

Among the 350 patients who underwent surgery, *n* = 283 had complete prescription data and were available for multivariable regression to predict average MME of opioid prescriptions ordered within 30 days of surgery. In preparation for multivariable analysis, we assessed normality assumptions of average MME distribution using the Kolmogorov-Smirnov test. The test revealed a non-normal distribution (K-S statistic = 0.497, *p* < 0.001), and upon visual inspection the distribution was highly positively skewed and bimodally distributed by average MME of opioids prescribed. To better understand causes of this bimodal distribution, we conducted logistic regression to predict membership in the lower average MME cohort (*n* = 250) or higher average MME cohort (*n* = 33). Surgical procedure was a highly significant predictor of cohort membership (Nagelkerke R square = 0.755, *p* < 0.001), as 27 of 33 (82%) patients in the higher average MME cohort underwent a PAO procedure. This is not surprising as these procedures are commonly more complex and often use different pain management regimens in the acute phase (i.e., patient controlled analgesia) compared to other hip procedures. Because these were deemed different populations for the purpose of this analysis, and because surgery type was the only predictor of average MME, we decided to exclude the PAO cohort from further multivariable analyses. This approach enabled us to better discern contributors beyond surgery type. Due to the small sample size of the PAO cohort, we were unable to perform further prediction modeling of MME in that cohort.

With PAO procedures excluded, and with remaining surgical procedures as a covariate in the model, the first model excluding iHOT-12 scores yielded two significant variables: surgery type and insurance type. Patients undergoing hip arthroscopy were prescribed opioid prescriptions with significantly higher average MME than arthroplasty (beta=-0.597, *p* < 0.001) and other procedures (beta=-0.675, *p* < 0.001). Patients with public insurance (Medicare/Medicaid) (beta=-0.255, *p* = 0.031) had prescriptions with significantly lower average MME than those with commercial or private insurance (Table 4). Results were largely unchanged when adding iHOT-12 scores to the model (Table 5). Patients undergoing arthroscopy again had prescriptions with significantly higher average MME than arthroplasty (beta=-0.623, *p* < 0.001) and other procedures (beta=-0.680, *p* < 0.001). Patients with public insurance (Medicare/Medicaid) (beta=-0.279, *p* = 0.019) or TRICARE/other government insurance (beta=-0.147, *p* = 0.039) received opioids with significantly lower average MME than those with commercial or private insurance.

**Table 4:** Multivariable linear regression analysis of predictors of average MME excluding iHOT-12 scores (*n* = 248)

Predictor	Beta coefficient	P-value
Age	-0.005	0.065
Female gender (Ref: Male)	0.018	0.762
Race (Ref: White)		
- Black/African American	-0.099	0.247
- Asian	0.262	0.107
- Not reported	0.137	0.438
- Other	0.046	0.817
Ethnicity (Ref: Not Hispanic/Latino)		
- Hispanic/Latino	-0.095	0.472
- Not reported	-0.059	0.776
Employment (Ref: Employed)		
- Not employed	0.117	0.215
- Student	-0.049	0.653
- Other/Disabled	0.045	0.710
Insurance (Ref: Private)		
- Public	-0.255	0.031
- TRICARE/other government	-0.133	0.061
- Other/Worker’s Compensation/Self pay	0.014	0.931
OSPRO-YF phenotype cluster (Ref: High Distress)		
- Negative pain coping	0.006	0.937
- Low distress	0.006	0.934
- Low self-efficacy and acceptance	-0.048	0.648
Type of surgery (Ref: Arthroscopy)		
- Arthroplasty	-0.597	< 0.001
- Other	-0.675	< 0.001

**Table 5:** Multivariable linear regression analysis of predictors of average MME including iHOT-12 scores (*n* = 248)

Predictor	Beta coefficient	P-value
Age	-0.005	0.063
Female gender (Ref: Male)	0.007	0.904
Race (Ref: White)		
- Black/African American	-0.113	0.187
- Asian	0.233	0.154
- Not reported	0.135	0.440
- Other	0.055	0.782
Ethnicity (Ref: Not Hispanic/Latino)		
- Hispanic/Latino	-0.098	0.455
- Not reported	-0.060	0.774
Employment (Ref: Employed)		
- Not employed	0.125	0.185
- Student	-0.028	0.797
- Other/Disabled	0.030	0.804
Insurance (Ref: Private)		
- Public	-0.279	0.019
- TRICARE/other government	-0.147	0.039
- Other/Worker’s Compensation/Self pay	-0.003	0.985
OSPRO-YF phenotype cluster (Ref: High Distress)		
- Negative pain coping	0.016	0.818
- Low distress	0.039	0.622
- Low self-efficacy and acceptance	-0.015	0.888
Type of surgery (Ref: Arthroscopy)		
- Arthroplasty	-0.623	< 0.001
- Other	-0.680	< 0.001
iHOT-12 scores	-0.002	0.158

In a final post-hoc analysis, we limited the analysis to patients undergoing hip arthroscopy only because (1) this was by far the most common procedure performed in our setting, and (2) we were interested if findings were robust after removing the variable of surgery type entirely. This analysis found two significant predictors when excluding iHOT-12 scores: age (beta=-0.009, *p* = 0.007; older patients had lower average MME) and insurance type, with those on public insurance (Medicare/Medicaid) being prescribed opioids with lower mean MME than those on commercial or private insurance (beta=-0.397, *p* = 0.006) (Table 6). Adding iHOT-12 scores did not meaningfully change the model, with age (beta=-0.009, *p* = 0.007) and insurance type remaining the only significant predictors (Table 7). Those on public insurance (Medicare/Medicaid) had lower mean MME than those on commercial or private insurance (beta=-0.423, *p* = 0.004). Gender, race, ethnicity, employment, OSPRO-YF phenotype assignment, and iHOT-12 scores were not significant unique predictors of mean MME in this sample.

**Table 6:** Multivariable linear regression analysis of predictors of average MME excluding iHOT-12 scores for arthroscopy sample only (*n* = 184)

Predictor	Beta coefficient	P-value
Age	-0.009	0.007
Female gender (Ref: Male)	0.080	0.236
Race (Ref: White)		
- Black/African American	-0.010	0.929
- Asian	0.294	0.103
- Not reported	0.197	0.345
- Other	0.091	0.660
Ethnicity (Ref: not hispanic/Latino)		
- Hispanic/Latino	-0.182	0.208
- Not reported	-0.087	0.688
Employment (Ref: Employed)		
- Not employed	0.134	0.265
- Student	-0.117	0.324
- Other/Disabled	0.134	0.358
Insurance (Ref: Private)		
- Public	-0.397	0.006
- TRICARE/other government	-0.113	0.156
- Other/Worker’s Compensation/Self pay	0.047	0.850
OSPRO-YF phenotype cluster (Ref: high distress)		
- Negative pain coping	-0.031	0.696
- Low distress	-0.096	0.271
- Low self-efficacy and acceptance	-0.093	0.490

**Table 7:** Multivariable linear regression analysis of predictors of average MME including iHOT-12 scores for arthroscopy sample only (*n* = 184)

Predictor	Beta coefficient	P-value
Age	-0.009	0.007
Female gender (Ref: Male)	0.067	0.329
Race (Ref: White)		
- Black/African American	-0.020	0.853
- Asian	0.270	0.136
- Not reported	0.199	0.338
- Other	0.097	0.642
Ethnicity (Ref: Not Hispanic/Latino)		
- Hispanic/Latino	-0.187	0.196
- Not reported	-0.090	0.678
Employment (Ref: Employed)		
- Not employed	0.148	0.218
- Student	-0.096	0.419
- Other/Disabled	0.120	0.411
Insurance (Ref: Private)		
- Public	-0.423	0.004
- TRICARE/other government	-0.128	0.113
- Other/Worker’s Compensation/Self pay	0.029	0.908
OSPRO-YF phenotype cluster (Ref: high distress)		
- Negative pain coping	-0.021	0.787
- Low distress	-0.068	0.455
- Low self-efficacy and acceptance	-0.066	0.634
iHOT-12 scores	-0.002	0.297

## Discussion

### Aim one: predictive model for risk of surgery in one year

We hypothesized that patients with higher levels of psychological distress, i.e., those belonging to the high distress phenotype, would have higher odds of undergoing surgery than those assigned to phenotypes characterized by lower levels of distress. Our hypothesis was supported; however, psychological distress was not a significant predictor when iHOT-12 scores were added to the risk of surgery model. Thus, when developing the most parsimonious model for predicting surgical risk in this cohort, information about psychological distress may be less important as a prognostic factor than information on function and quality of life measured by the iHOT-12. One potential explanation for this finding is that the iHOT-12 captures multiple dimensions of the patient, including symptoms, function, lifestyle, physical activities, employment, socioeconomic status, and emotional wellbeing [27]. Level of psychological distress is likely reflected in these characteristics. Although iHOT-12 and OSPRO-YF scores are correlated, they did not meet multicollinearity thresholds that would preclude both being considered together in a statistical model. Given that iHOT-12 captures dimensions related to function, socioeconomic status, and well-being, it is not entirely surprising that iHOT-12 would be a better predictor of surgery, considering the complex factors that weigh on the decision-making process for patients. Our results suggest that factors that tend to covary with psychological distress, such as age, race, and iHOT-12 scores, provide a better indication of surgical risk than information captured by the OSPRO-YF. Importantly, these findings do not suggest that addressing psychological distress provides no clinical value in this cohort. They only suggest that assignment of psychological phenotype using OSPRO-YF may not add value as a prognostic factor when more comprehensive information, such as provided by the iHOT-12, is available.

Younger patients in this cohort were more likely to undergo surgery, and that finding was robust regardless of other variables in the models, such as gender, race, ethnicity, insurance, and psychological distress. Surgical candidacy, while informed by objective measures such as imaging and physical examination, is still likely influenced by subjective criteria, as perceived superior patient fitness to withstand surgery and tolerate postoperative rehabilitation among younger patients may influence treatments offered to patients. However, other studies have highlighted comparable postoperative joint pain, function, and quality of life in patients across the age spectrum after hip and knee arthroplasty [41]. If surgery is deemed unsuitable for older patients, providers are responsible for weighing the potential benefits and harms of surgery, engaging in shared decision-making, and gauging patients’ interest in a variety of non-operative pain management treatment offerings [42–45]. Interventions that address psychological distress may be especially critical in non-operative patients to prevent further disability.

In the model excluding iHOT-12 scores, insurance was also a significant predictor of hip surgery, specifically for patients with TRICARE/other government insurance. This subset consisted of 120 patients covered by TRICARE insurance, which includes active-duty military service members and retirees, while Medicare and Medicaid recipients were classified separately in the public insurance category. TRICARE recipients may present with unique needs for surgical intervention based on a higher prevalence of osteoarthritis and joint pain, more advanced hip pathology, or intense physical activity requirements [46–48]. Prior studies have found associations between TRICARE recipients, increased healthcare utilization, and higher treatment costs for combat-related orthopaedic injuries vs. non-combat related injuries [49]. Similarly, Dalton et al. (2023) found that TRICARE recipients, especially those with combat-related injuries, were at risk for mental health conditions, underscoring the importance of preventing delays in orthopaedic and psychological evaluation [50].

In the model including iHOT-12 scores, our analyses found that self-identified Black and African American patients had lower odds of undergoing hip surgery. Disparities in access to musculoskeletal care are well-documented (51–52), and differences in treatment selection have also been observed for Black populations seeking management for knee pathology [53]. Similarly, mistrust of health care systems and providers among minority populations also may play a pivotal role in whether patients elect to undergo surgery [54]. Social factors such as access to postoperative rehabilitation care and high-quality physical therapy, transportation barriers, and insurance also could influence a patient’s decision to undergo surgery [55–58]. Ultimately, more research is required to understand why hip surgery was less commonly observed in this population and how to eliminate barriers to orthopaedic treatment.

### Aim two: predictive model for mean MME of opioid prescriptions

The predictive models for mean MME of opioid prescriptions received within 30 days of surgery provided novel insights into opioid prescribing patterns among patients with hip pain and pathologies. Interestingly, risk for higher average MME was associated with factors that are largely non-modifiable: age, insurance, and surgery type. Surgery type and insurance type were consistent predictors of average MME, as patients who underwent hip arthroscopy and those with private insurance had greater odds of being prescribed opioids with higher average MME compared to arthroplasty patients and Medicare/Medicaid/TRICARE recipients, respectively. Moreover, our results suggest that postoperative pain management and average MME may be more closely associated with provider behavior and external factors (e.g., access to care and insurance coverage) than patient-level factors dictating need. Nonetheless, older adults, active-duty service members, veterans, and those with lower socioeconomic status receiving postoperative opioids remain crucial populations to target in risk reduction efforts given several prior studies have demonstrated these populations are disproportionately impacted by the opioid crisis [17, 59–62].

We did not find OSPRO-YF or iHOT-12 information to meaningfully improve prediction of opioids prescribed in any analysis. However, these findings should be confirmed in larger samples. Patients in the PAO subgroup received opioid prescriptions with greater MME. Given the greater intensity and complexity of the PAO procedure versus arthroscopy and arthroplasty, patients who underwent PAO with concomitant hip arthroscopy may have had increased pain management requirements than those who underwent less invasive procedures such as hip arthroscopy or arthroplasty alone. Similarly, providers must pay careful attention to the route of administration of opioids, as all patients who underwent PAO in this cohort had received a postoperative patient-controlled analgesia epidural. Patients undergoing invasive orthopaedic procedures benefit from close monitoring and follow-up in the postoperative period to reduce the risk of opioid overconsumption, dependence, and diversion [63].

### Limitations

This study has limitations that warrant discussion. First, our study assessed risk of surgery within one year of evaluation. Consequently, our results may not capture the surgical risk of patients at all stages of disease progression, and some patients in this cohort may elect to undergo surgery in the future. Second, it was not possible to analyze patients’ actual opioid consumption given the inability to corroborate patient adherence; instead, opioids prescribed by providers were used as a proxy. This is a common challenge of opioid-related research that uses retrospective EHR data. Third, we chose a short observation window of 30 days after surgery for the opioid prescribing data and acknowledge that postoperative opioid prescribing may extend beyond this period for select patients. Nonetheless, this analysis adds value to decision-making given the increasing awareness of the critical nature of the first 30 days after surgery as a risk factor for chronic opioid use. In addition, our analysis did not draw on patients’ prior or preoperative opioid histories, as associations between preoperative and postoperative opioid consumption among orthopaedic patients are already well-established [64–67]. Prior or preoperative opioid use could have influenced the postoperative pain management requirements of our cohort. Approximately 19% (*n* = 67) of patients who underwent surgery had incomplete medication data and were excluded from multivariable analysis. However, we compared baseline demographic and health-related data and found no significant differences between those with and without complete data. Also, as this was a retrospective study, we were unable to account for health care utilization that occurred outside of our system. Additionally, we were unable to conduct PAO-specific multivariable analyses due to the small sample size of that cohort. Given the high opioid prescribing in the PAO cohort, future studies are warranted in larger samples to better understand factors that account for variability in prescribing. Hip pathology diagnosis was not included as a surgical predictor in analyses in large part because we had limited variability in the diagnostic indications for which patients sought care in our hip preservation clinic. Diagnosis was indirectly considered in the MME analyses by inclusion of surgery type. Finally, we were unable to account for some injuries that could result in higher than average risk of surgery or opioid prescribing, such as injuries from motor vehicle collisions.

## Conclusions

This study generated insights on the relationships between psychological distress, surgical risk and decision-making, and opioid prescribing patterns among patients with hip pain. Psychological distress was associated with risk of orthopaedic surgery when controlling for patient demographics. However, consideration of psychological distress phenotypes does not appear to add predictive value for surgical risk or opioid prescribing outcomes when considered alongside other clinical and patient-reported information, such as iHOT-12 scores, age, insurance, and surgery type. Modifiable characteristics like function, quality of life, and psychological distress may be important targets for treatment and rehabilitation in this cohort.

## Data Availability

The datasets used and/or analyzed during the current study are available from the corresponding author on reasonable request. Ensuring participant confidentiality is of the utmost importance to the authors.

## Abbreviations

OSPRO-YF: Optimal Screening for Prediction of Referral and Outcome Yellow Flag Assessment Tool
iHOT-12: International Hip Outcome Tool
MME: Morphine milligram equivalents
PAO: Periacetabular osteotomy
